# Quantitative ultrasound assessment of fatty infiltration of the rotator cuff muscles using backscatter coefficient

**DOI:** 10.1186/s41747-024-00522-5

**Published:** 2024-10-22

**Authors:** Marco Toto-Brocchi, Yuanshan Wu, Saeed Jerban, Aiguo Han, Michael Andre, Sameer B. Shah, Eric Y. Chang

**Affiliations:** 1 Department of Radiology, University of California, San Diego, CA USA; 2 Department of Radiology, Università Degli Studi Di Milano, Milan, Italy; 3 Research Service, VA San Diego Healthcare System, San Diego, CA USA; 4 Department of Bioengineering, University of California, San Diego, CA USA; 5 Department of Biomedical Engineering and Mechanics, Virginia Polytechnic Institute and State University, Blacksburg, VA USA; 6 Department of Orthopaedic Surgery, University of California, San Diego, CA USA; 7 Radiology Service, VA San Diego Healthcare System, San Diego, CA USA

**Keywords:** Magnetic resonance imaging, Muscular atrophy, Rotator cuff, Shoulder, Ultrasonography

## Abstract

**Background:**

To prospectively evaluate ultrasound backscatter coefficients (BSCs) of the supraspinatus and infraspinatus muscles and compare with Goutallier classification on magnetic resonance imaging (MRI).

**Methods:**

Fifty-six participants had shoulder MRI exams and ultrasound exams of the supraspinatus and infraspinatus muscles. Goutallier MRI grades were determined and BSCs were measured. Group means were compared and the strength of relationships between the measures were determined. Using binarized Goutallier groups (0–2 *versus* 3–4), areas under the receiver operating characteristic curves (AUROCs) were calculated. The nearest integer cutoff value was determined using Youden’s index.

**Results:**

BSC values were significantly different among most Goutallier grades for the supraspinatus and infraspinatus muscles (both *p* < 0.001). Strong correlations were found between the BSC values and Goutallier grades for the supraspinatus (τ_b_ = 0.72, *p* < 0.001) and infraspinatus (τ_b_ = 0.79, *p* < 0.001) muscles. BSC showed excellent performance for classification of the binarized groups (0–2 *versus* 3–4) for both supraspinatus (AUROC = 0.98, *p* < 0.0001) and infraspinatus (AUROC = 0.98, *p* < 0.0001) muscles. Using a cutoff BSC value of −17 dB, sensitivity, specificity, and accuracy for severe fatty infiltration were 87.0%, 90.0%, and 87.5% for the supraspinatus muscle, and 93.6%, 87.5%, and 92.7% for the infraspinatus muscle.

**Conclusion:**

BSC can be applied to the rotator cuff muscles for assessment of fatty infiltration. For both the supraspinatus and infraspinatus muscles, BSC values significantly increased with higher Goutallier grades and showed strong performance in distinguishing low *versus* high Goutallier grades.

**Relevance statement:**

Fatty infiltration of the rotator cuff muscles can be quantified using BSC values, which are higher with increasing Goutallier grades.

**Key Points:**

- Ultrasound BSC measurements are reliable for the quantification of muscle fatty infiltration.
- BCS values increased with higher Goutallier MRI grades.
- BCS values demonstrated high performance for distinguishing muscle fatty infiltration groups.

**Graphical Abstract:**

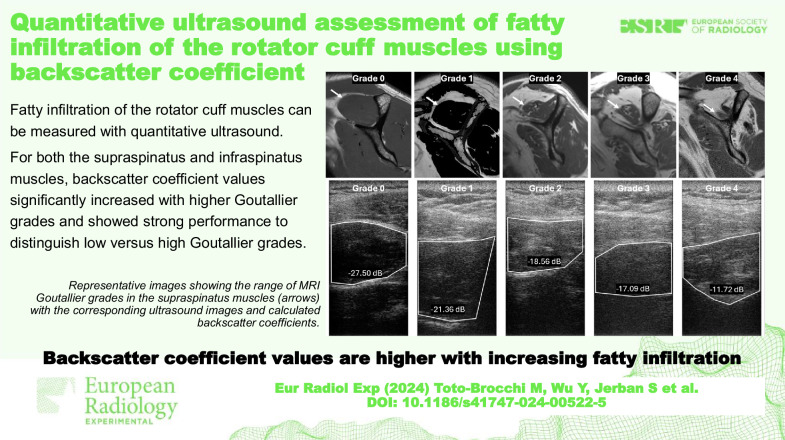

## Background

Rotator cuff tendon tears are common and associated with functional and structural deterioration of the glenohumeral joint [1]. Rotator cuff tendon repair leads to improved outcomes, particularly if the tendon heals [2]. However, numerous studies have shown poorer functional outcomes and higher retear rates in patients with rotator cuff muscle abnormalities, including atrophy and fatty infiltration [3]. The severity of myosteatosis has recently emerged as one of the most important prognostic factors in the successful operative management of rotator cuff tears [4].

Magnetic resonance imaging (MRI) is considered a reference standard modality for the clinical evaluation of rotator cuff muscle status, and the five-grade Goutallier classification is the most widely used method for the assessment of fatty infiltration [5]. Interest in using ultrasound imaging for this purpose has increased over the years since ultrasound is lower in cost, faster, and more comfortable for patients [5–7]. Modified three-grade versions of the Goutallier classification have been proposed and used with ultrasound imaging [8, 9]. However, the limitations of MRI- and ultrasound-based Goutallier classifications are the same in that they are not fully quantitative and reliability is suboptimal, generally ranging from fair to substantial [9–11].

Quantitative ultrasound methods, including those based on analysis of raw radiofrequency data, are more objective, system-independent, and provide information about tissue structure and disease status [12, 13]. The backscatter coefficient (BSC) is one such quantitative ultrasound parameter that describes internal scattering structures in a medium [14], analogous to echogenicity assessed qualitatively. While B-mode image evaluation is highly dependent on overlying tissue attenuation (*i.e*., composition and varying depth), total attenuation from the skin surface to the region of interest is compensated in the BSC measurement [13]. BSC was recently used on rotator cuff muscles and shown to be repeatable and reproducible across different operators and ultrasound imaging platforms [15]. Although the quantitative ultrasound measurement of BSC was shown to be more reliable than other measures made on routine B-mode images [15], the utility of BSC for quantifying rotator cuff muscle abnormalities across the spectrum of disease remains unknown.

The purpose of this study was to prospectively evaluate the BSCs of the supraspinatus and infraspinatus muscles and compare them with the Goutallier classification on MRI.

## Methods

### Study population

This study was approved by our Institutional Review Board and all participants provided and signed a statement of informed consent. Between February and March of 2024, all patients presenting to our radiology department for shoulder MRI exams were screened for this study. Patients who declined to participate and those with clinical MRI indications for tumors or infection were excluded. All others were included regardless of precise history (*e.g.*, pain or trauma). In addition, trainee volunteers from the department were invited to participate so long as they could undergo the imaging techniques, regardless of history or symptoms.

### Image acquisition

MRI was performed using a dedicated shoulder coil on one of three scanners, including 1.5-T (Signa Artist, GE Healthcare, Milwaukee, WI, USA) and 3-T (Skyra Fit, Siemens Healthineers, Erlangen, Germany or MR750, GE Healthcare, Milwaukee, WI, USA) systems. The imaging protocols included fat-suppressed axial intermediate-weighted, fat-suppressed oblique coronal T2-weighted, oblique coronal T1-weighted, fat-suppressed oblique sagittal T2-weighted, and oblique sagittal T1-weighted sequences.

Ultrasound imaging was performed using a linear probe with a clinical ultrasound machine (14L5, S2000, Siemens Healthineers, Erlangen, Germany) and beam-formed radiofrequency signals were acquired. One of two operators performed the exam (E.Y.C., a musculoskeletal radiologist with 13 years of musculoskeletal ultrasound experience, or M.T.-B., a radiology resident with 4 years of general ultrasound training and 3 weeks of additional shoulder ultrasound training by E.Y.C.). The supraspinatus muscle was imaged on a short axis approximately 1 inch medial to the acromion and the infraspinatus muscle was inferior to the scapular spine, also on short axis. Three repeated measurements were made for each muscle. To facilitate the speed of imaging, the same imaging preset was used for all participants, including depth, focus, time gain compensation, and receiver gain. Data was also obtained from a calibrated, commercially available, homogeneous, tissue-mimicking phantom containing 117GU Zerdine formulation (Sun Nuclear, Norfolk, VA, USA).

### Image analysis

Images from MRI examinations were independently graded by E.Y.C. and M.T.-B. following the protocol described by Fuchs et al [16, 17]. Specifically, the most lateral oblique sagittal T1-weighted image on which the scapular spine was in contact with the scapular body was used and the presence of fatty infiltration was graded according to the semiquantitative scale described by Goutallier et al [17]. Grade 0 is completely normal without any fatty streaks, grade 1 contains some fatty streaks, grade 2 contains more muscle than fat, grade 3 contains equal amounts of fat and muscle, and grade 4 contains more fat than muscle. Discrepancies between gradings were resolved in consensus and a final Goutallier grade was assigned for the remainder of the analyses. Supraspinatus muscle atrophy was also measured on the same T1-weighted image by M.T.-B. using the occupation ratio as described by Thomazeau et al [18]. The occupation ratio is the ratio between the cross-sectional areas of the supraspinatus muscle divided by that of its fossa. RadiAnt DICOM Viewer (v. 2020.2.3, Medixant, Poznan, Poland) was used for clinical MRI analysis.

Fatty infiltration was evaluated on the B-mode ultrasound images using the method described by Wall et al [9], which was modified from the scale previously described by Strobel et al [11]. Specifically, echogenicity (assessed relative to overlying muscle with 0 as isoechoic, one as slightly increased, and two as markedly increased) and architecture (visibility of intramuscular tendon and pennation pattern with 0 as clearly visible, one as partially visible, and two as not discernable) of each muscle were examined, with use of three-point scales. The grades for echogenicity and architecture were averaged to determine a single grade (0–2) for each muscle.

Quantitative ultrasound images were analyzed using a standardized graphical user interface [19], which calculated the integral BSC between 5 MHz and 10 MHz using the reference phantom method [20]. The entire rotator cuff muscle on the ultrasound image was manually outlined, including the epimysium (by M.T.-B.). A separate region of interest was placed over all of the overlying tissues and frequency-dependent attenuation was corrected using the spectral log difference method [21, 22]. A diagram illustrating the processing steps required to compute the BSC is shown in Supplemental Fig. S1. For increased stability of calculated values, the graphical user interface automatically subdivides the region of interest into 75% overlapping subregions of interest, each with dimensions of 15 wavelengths in the lateral and axial directions, in accordance with recommendations from the literature [21, 22]. Typical post-processing times with the graphical user interface are less than 10 s per image. The three repeated measurements for each muscle were averaged and considered as a single measurement for the remainder of the analyses. For an assessment of reliability, ten participants were randomly selected and BSC measurements were independently measured by a second reader (E.Y.C.) in the same manner.

### Statistical analysis

The Shapiro–Wilk test was used to assess data normality distribution and the appropriate statistical test was chosen based on the data. Descriptive statistics were performed. Inter-observer reliability of supraspinatus and infraspinatus MRI Goutallier grading and ultrasound determination of fatty infiltration was assessed using Cohen ĸ), which was interpreted as follows: 0.0–0.2, slight; 0.21–0.4, fair; 0.41–0.6, moderate; 0.61–0.80, substantial; 0.81–1.0, and almost perfect [23]. Two-way mixed intraclass correlation (ICC) coefficients were used to assess inter-observer reliability for BSC measurements. A one-way analysis of variance (ANOVA) was performed to compare the BSC differences between various Goutallier grade groups. *Post hoc* comparisons between each Goutallier group were performed using the Tukey honest significant difference test.

The strength of the relationships between BSC, Goutallier grade, and occupation ratio was assessed using Kendall’s tau-b (τ_b_) or Pearson correlation, as appropriate. All correlations were interpreted as: 0.0–0.1, negligible; 0.1–0.39, weak; 0.4–0.69, moderate; 0.7–0.89, strong; 0.9–1.0, very strong [24].

Goutallier groups were binarized as 0–2 *versus* 3–4 as suggested in a systematic review [4], and the area under the receiver operating characteristic curves (AUROCs) was calculated. AUROCs were interpreted as: 0.0–0.59, failed model discrimination; 0.6–0.69, poor; 0.7–0.79, fair; 0.8–0.89, good; 0.9–1.0, excellent [25]. Bootstrapping of the BSC data was performed in Python v3.10.12, where the same number of subjects as that in the given muscle groups were randomly selected with replacement. The AUROCs were then computed using the bootstrapped sample and the processes were repeated 1,000 times. The nonparametric 95% CIs for AUROCs were computed based on the 2.5th and 97.5th percentiles of the ordered distribution of AUROCs from the 1,000 samples. In addition, the point where Youden’s index was maximum (optimal sensitivity and specificity -1) was taken into account as the cut-off values [26]. The performance of the nearest integer cut-off value was also evaluated, as this would facilitate clinical translation.

An a priori power analysis was not conducted; however, a *post hoc* power analysis was performed (G*Power 3.1.9.7) using our study results, and this study had a power of 1.0 to detect a significant difference and an α error (the probability of yielding a type-I error) equal to 0.05. A *p*-value less than 0.05 was considered to indicate a significant difference. All statistical analyses were performed with IBM SPSS Statistics for Windows version 28.0 (IBM, Armonk, NY, USA) except as indicated above.

## Results

Population characteristics are shown in Table 1. Forty-six patients met the initial inclusion criteria and 14 trainee volunteers agreed to participate. The number of days between the MR and ultrasound imaging exams was 4.5 ± 7.8 days (mean ± standard deviation). Four participants who were scanned were ultimately excluded since the ultrasound image quality was deemed too low to outline the rotator cuff muscle boundaries due to attenuation (the mean body mass index of these excluded participants was 35.3 kg/m^2^). In total, 56 participants were included (54 males and 2 females, aged 49.8 ± 17.5 years).

**Table 1:** Population characteristics (*n* = 56)

Characteristics	Number or mean ± standard deviation
Age	49.8 ± 17.5 years
Male sex	54
Female sex	2
Days between MRI and ultrasound exams	4.5 ± 7.8 days
Goutallier grade 0	26 (supraspinatus), 16 (infraspinatus)
Goutallier grade 1	14 (supraspinatus), 21 (infraspinatus)
Goutallier grade 2	6 (supraspinatus), 10 (infraspinatus)
Goutallier grade 3	3 (supraspinatus), 3 (infraspinatus)
Goutallier grade 4	7 (supraspinatus), 6 (infraspinatus)

*MRI* Magnetic resonance imaging

**Fig. 1:**
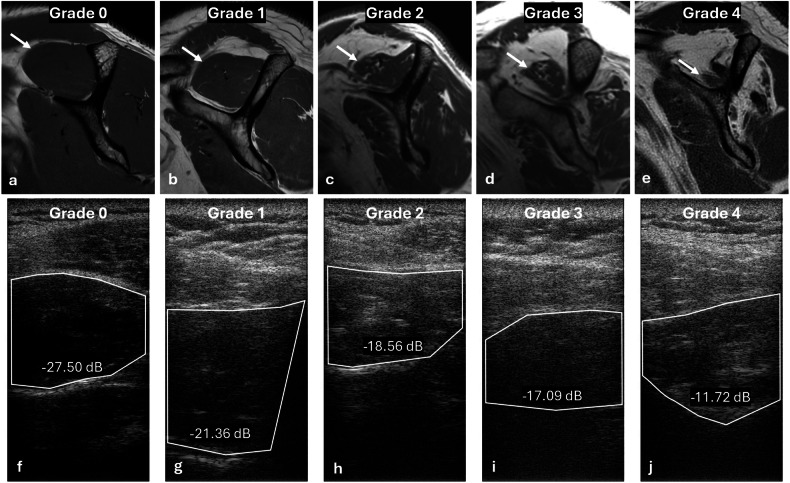
Representative images showing the range of MRI Goutallier grades (**a**–**e**) in the supraspinatus muscles (arrows) with the corresponding ultrasound images (**f**–**j**) and calculated backscatter coefficients from the regions of interest. Notice that the backscatter coefficient values increase with higher grades, however, the echogenicity on the B-mode images does not follow the same trend. B-mode echogenicity is determined by a variety of variables, including depth, whereas overlying tissue attenuation is corrected with quantitative ultrasound. MRI, Magnetic resonance imaging

Interobserver reliability for MRI Goutallier grading was substantial for the supraspinatus muscles (ĸ = 0.67, *p* < 0.001) and moderate for the infraspinatus muscles (ĸ = 0.51, *p* < 0.001). Inter-observer reliability for ultrasound grading of fatty infiltration was moderate for the supraspinatus muscles (ĸ = 0.56, *p* < 0.001) and fair for the infraspinatus muscles (ĸ = 0.43, *p* < 0.001). Inter-observer reliability for BSC measurements was near perfect (ICC = 0.99, *p* < 0.001 for both supraspinatus and infraspinatus muscles). Representative MR and ultrasound images are shown in Fig. 1.

**Fig. 2:**
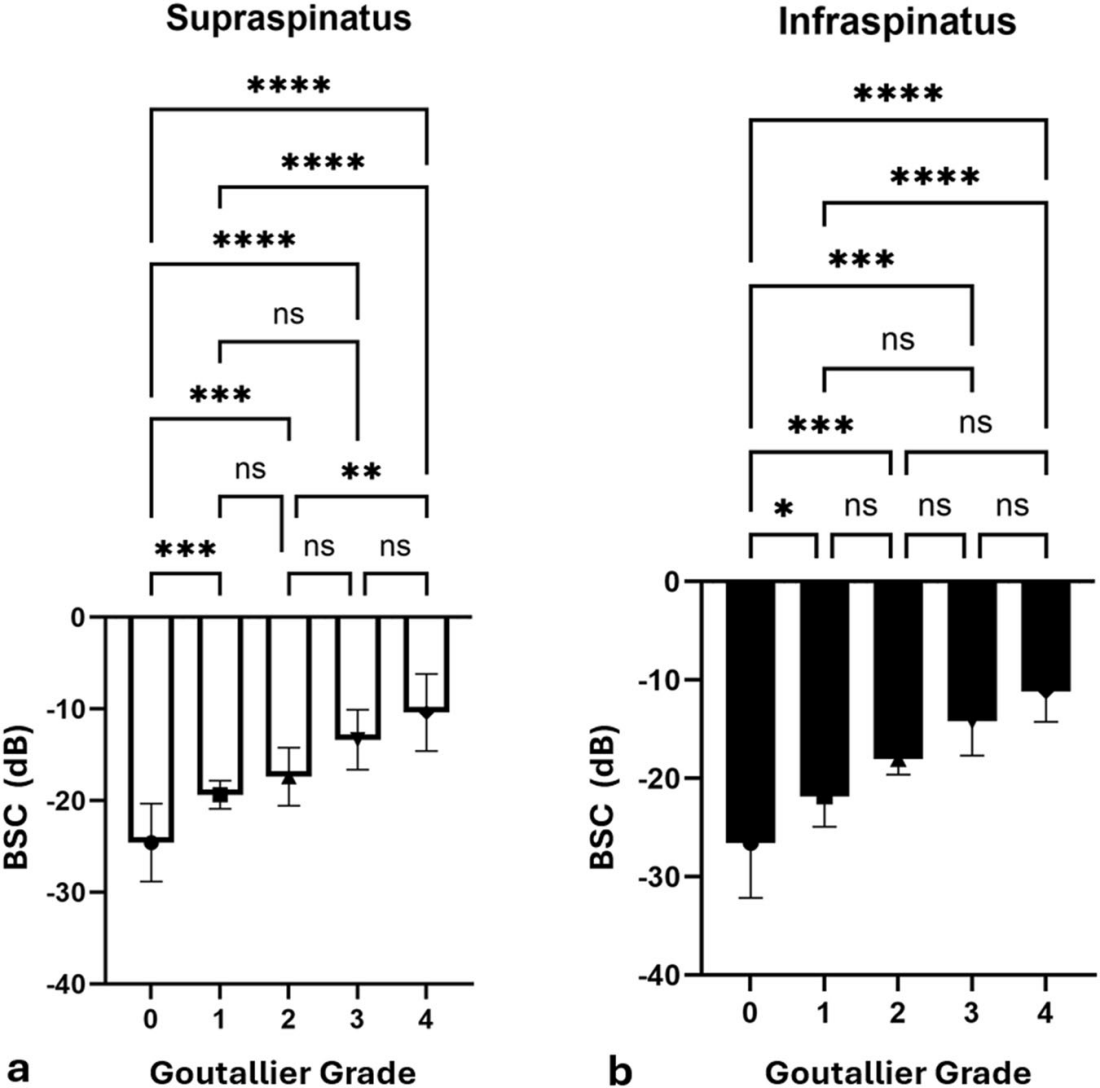
Bar plots showing the backscatter coefficient *versus* Goutallier grade for the supraspinatus (**a**) and infraspinatus (**b**) muscles. *p < 0.05, **p < 0.01, ***p < 0.001, ns, Not significant; BSC, Backscatter coefficient

BSC values were significantly different among most Goutallier grades for the supraspinatus and infraspinatus muscles (both *p* < 0.001) (Fig. 2 and Table 2). For the supraspinatus muscle, BSC values for each Goutallier grade were: -24.57 ± 4.24 for grade 0, -19.34 ± 1.53 for grade 1, -17.39 ± 3.17 for grade 2, -13.35 ± 3.26 for grade 3, and -10.38 ± 4.19 for grade 4. For the infraspinatus muscle, BSC values for each Goutallier were: -30.07 ± 4.05 for grade 0, -21.89 ± 2.34 for grade 1, -17.32 ± 2.85 for grade 2, -13.72 ± 4.27 for grade 3, and -11.59 ± 3.25 for grade 4.

**Table 2:** Rotator cuff backscatter coefficient values (mean ± standard deviation) for each MRI Goutallier grade

	MRI Goutallier grade				
	0	1	2	3	4
Supraspinatus	-24.57 ± 4.24	-19.34 ± 1.53	-17.39 ± 3.17	-13.35 ± 3.26	-10.38 ± 4.19
Infraspinatus	-30.07 ± 4.05	-21.89 ± 2.34	-17.32 ± 2.85	-13.72 ± 4.27	-11.59 ± 3.25

*MRI* Magnetic resonance imaging

**Fig. 3:**
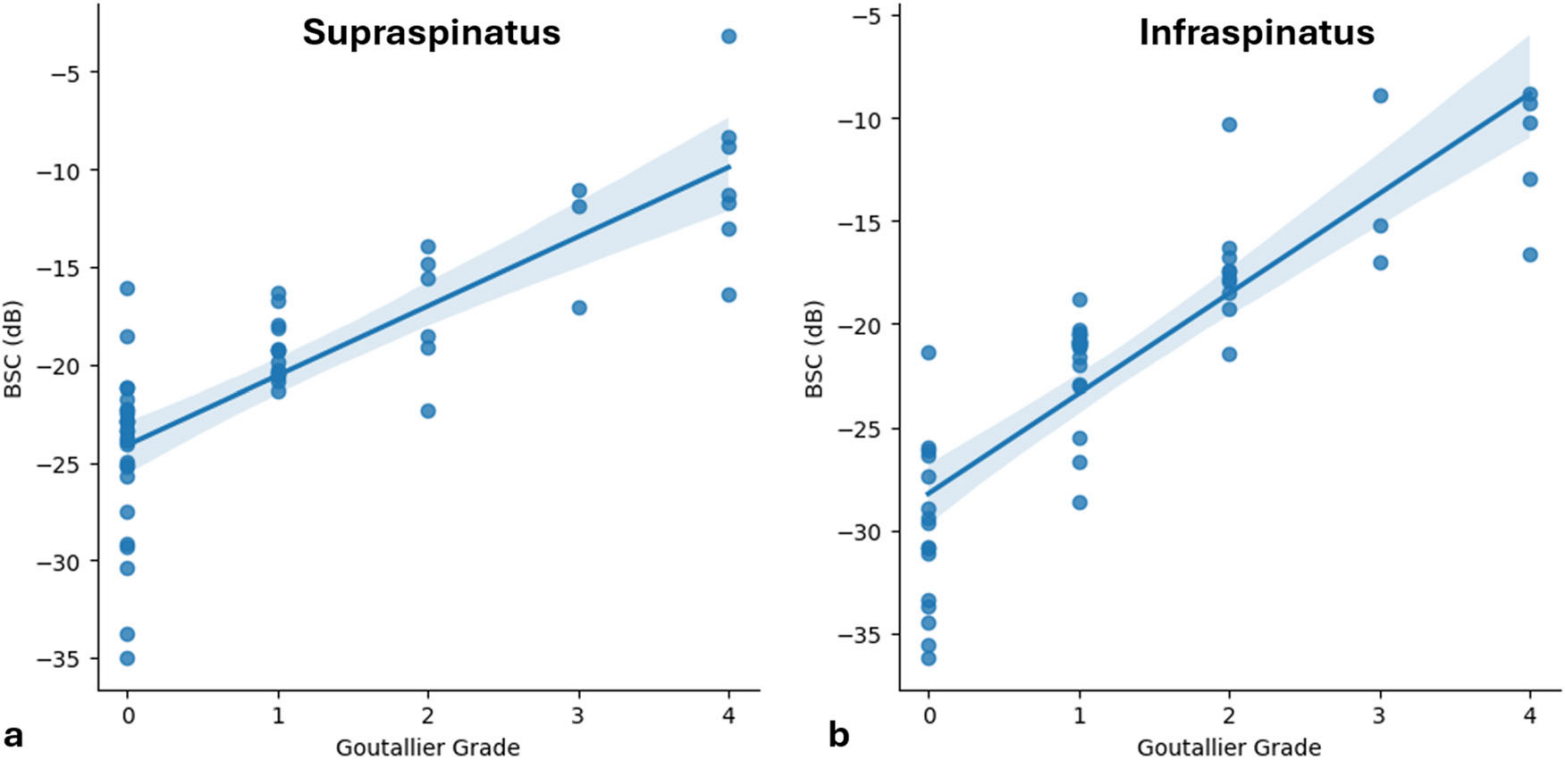
Scatter plots between backscatter coefficient and Goutallier grade for both the supraspinatus (**a**, τ_b_ = 0.72) and infraspinatus (**b**, τ_b_ = 0.79) muscles with regression lines and 95% confidence intervals. BSC, Backscatter coefficient

**Fig. 4:**
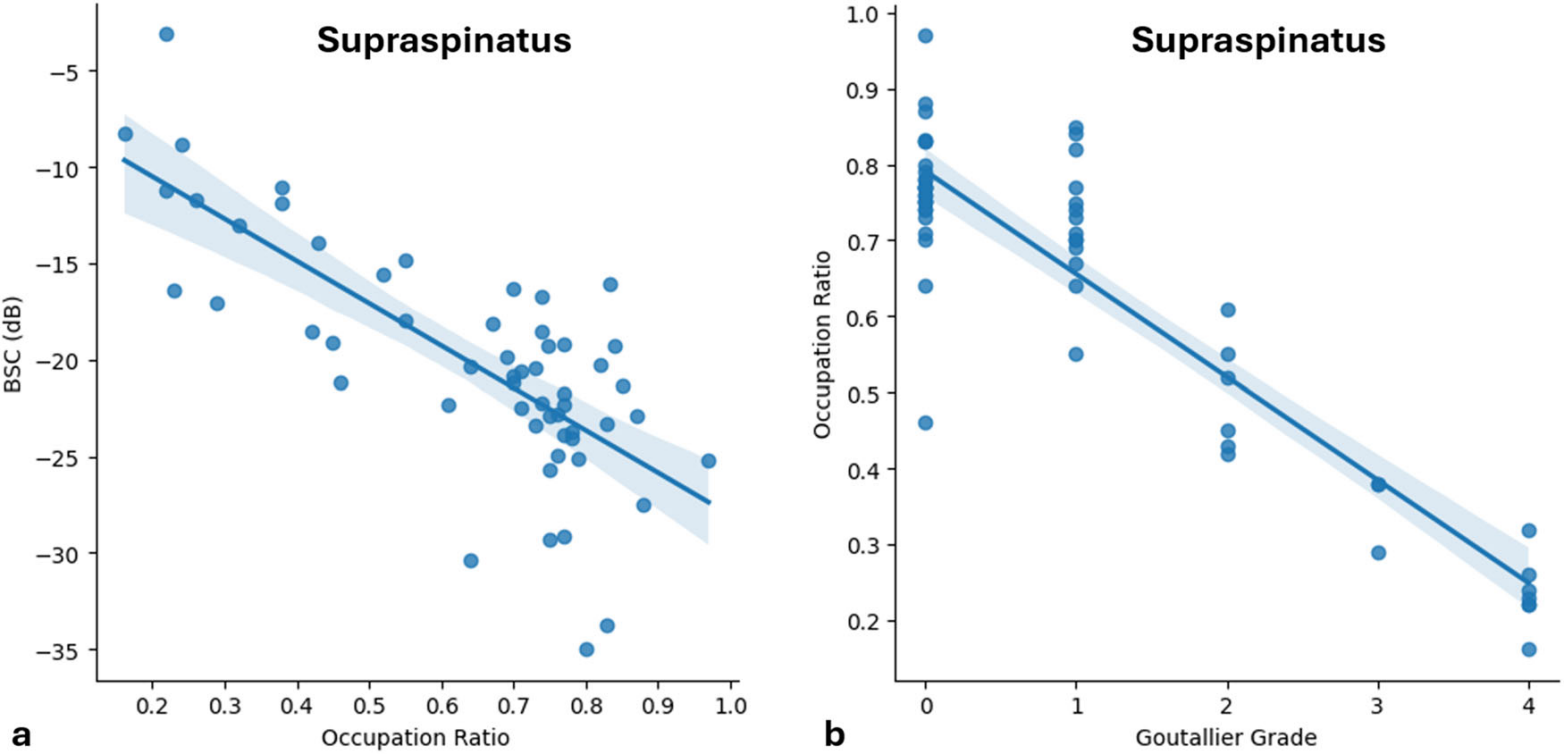
Scatter plots between backscatter coefficient and occupation ratio (**a**, *r* = -0.75) and occupation ratio and Goutallier grade (**b**, τ_b_ = -0.66) for the supraspinatus muscle with regression lines and 95% confidence intervals. BSC, Backscatter coefficient

Strong correlations were found between the BSC values and Goutallier grades for the supraspinatus (Kendall’s τ_b_ = 0.72, *p* < 0.001) and infraspinatus (Kendall’s τ_b_ = 0.79, *p* < 0.001) muscles (Fig. 3). For the supraspinatus muscle, a strong negative correlation was found between the BSC values and the occupation ratio (Pearson correlation coefficient = -0.75, *p* < 0.001) and a moderate negative correlation was found between the occupation ratio and Goutallier grade (Kendall’s τ_b_ = -0.66, *p* < 0.001) (Fig. 4).

**Fig. 5:**
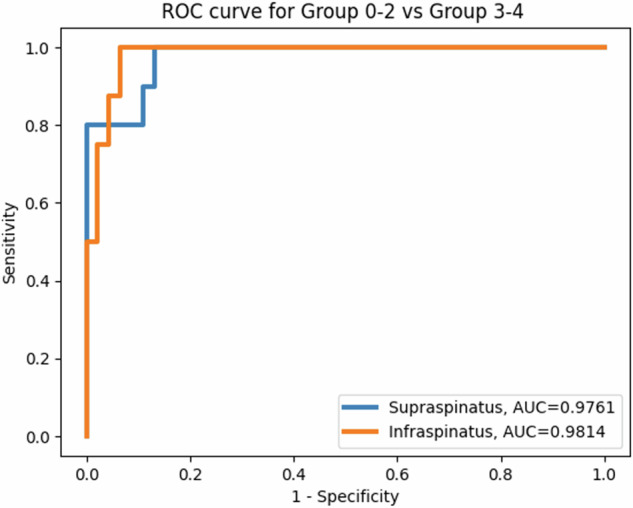
Receiver operating characteristic (ROC) curves for the supraspinatus and infraspinatus muscles. AUROC, Area under the curve

BSC showed excellent performance for classification of the binarized groups (0–2 *versus* 3–4) for both supraspinatus (AUROC = 0.98, *p* < 0.0001) and infraspinatus (AUROC = 0.98, *p* < 0.0001) muscles (Fig. 5). The histogram distributions of these groups are shown in Supplemental Fig. S2 and bootstrapped receiver operating characteristics analysis results are shown in Supplemental Fig. S3. For the supraspinatus muscle, a cut-off BSC value of -17.09 dB showed sensitivity, specificity, and accuracy of 100.0%, 87.0%, and 87.5%, respectively. For the infraspinatus muscle, a cut-off BSC value of -17.02 dB showed sensitivity, specificity, and accuracy of 100%, 93.6%, and 92.7%, respectively.

If a cutoff BSC value of -17.0 dB was used, sensitivity, specificity, and accuracy for the supraspinatus muscle would be 90.0%, 87.0%, and 87.5%, respectively, and sensitivity, specificity, and accuracy for the infraspinatus muscle would be 87.5%, 93.6%, and 92.7%, respectively.

## Discussion

In this study, we utilized BSC measurements to evaluate rotator cuff muscles across a spectrum of diseases as determined by MRI, a widely accepted reference standard. We found that BSC values were significantly different for several of the Goutallier grades and increased monotonically with higher grades. In addition, a recent systematic review including nine studies suggested that pre-operative MRI Goutallier grades may be best evaluated as two groups (Goutallier grades 0–2 and 3–4) [4]. Although no significant differences were shown between retear rates of preoperative Goutallier grades 0 through 2, when evaluating the binarized groups, grades 3–4 showed more than double the tear rates of grades 0–2 for both supraspinatus and infraspinatus muscles [4]. The results of our current study show that BSC values can distinguish between these two groups with accuracies in the range of 88–93%.

Evaluation of the muscle with ultrasound has a number of benefits compared with routine MRI assessments, including costs that are nearly half [27] and acquisition speeds of seconds compared with many minutes. Prior research has suggested that qualitative B-mode image evaluation of the rotator cuff muscles yields accuracies ranging from 72%–85% for substantial muscle atrophy [11]. However, reliability is suboptimal, as shown in our study (κ = 0.43–0.56), as well as others (*e.g*., κ = 0.50–0.71 [9, 11, 28]). Our results suggest that even higher accuracies can be achieved using quantitative BSC, which is a continuous measure that has consistently shown high accuracy, precision, repeatability, or reproducibility as applied to a variety of organs, including the liver [14], nerves [29], and rotator cuff muscles [15]. In comparison, we found only moderate to substantial reliability of Goutallier grading on MRI (κ = 0.51–0.67), in keeping with values that have been reported in the literature (*e.g*., 0.41 [30], 0.59 [31], and 0.66–0.82 [32]).

While Goutallier grade measures muscle fatty infiltration, the occupation ratio is a measure of total muscle volume loss or atrophy. Fatty infiltration and atrophy are interdependent, likely representing different manifestations of the same disease process, but not perfectly correlated [8]. The occupation ratio measurement can be performed on MRI or routine ultrasound, with a good correlation between the two modalities [8]. One study used an MRI-based deep-learning framework to assist in the quantification of fatty infiltration and compared it with the occupation ratio, showing a moderate negative correlation (ρ  =  -0.75) [33]. Notably, this is the same correlation result we achieved with BSC and occupation ratio (ρ = -0.75).

Although B-mode evaluation is the mainstay of ultrasound imaging in clinical practice, B-mode images are sensitive to varying scanner settings such as beam focus, frequency, transmit and receiver gains, time gain compensation, as well as proprietary postprocessing techniques that vary by vendors. These variables hinder comparisons of intensities and textures between images, patients, and scanners. In contrast, quantitative ultrasound BSC-based measurements allow for repeatable and reproducible comparisons to be made between patients, operators, and different ultrasound systems regardless of scanner settings [15]. There are drawbacks, however, most notably the requirement for raw data to be acquired from the scanner and data to be collected from a calibrated reference phantom. Offline processing must also be performed, at least until online processing becomes commercially available.

Our study has limitations. First, the majority of participants in our study were male, but this reflects the cohort of patients at our institution (approximately 90% of United States Veterans are male). Second, four of our participants were excluded post hoc due to excessive attenuation related to obesity. Scanning with a lower frequency transducer could have been possible but was not performed because the difficulties in outlining the muscles on the degraded images were not recognized prospectively. Fourth, standard MRI techniques were used as the reference standard in this study. Stronger reference standards exist, including quantitative MRI and histology, which may be beneficial to compare with in future studies. Fifth, a variety of additional quantitative ultrasound parameters exist which were not included in our study [12]. Finally, our study included a relatively small sample size, which limits finer diagnostic power. Although post hoc power testing showed that we were adequately powered to detect a significant difference with the one-way ANOVA test, we were likely underpowered for pairwise post hoc testing using the Tukey honest significant difference test.

In conclusion, ultrasound BSC measurements can be applied to the rotator cuff muscles for assessment of fatty infiltration and atrophy. For both the supraspinatus and infraspinatus muscles, BSC values significantly increased with higher Goutallier grades and showed strong performance in distinguishing between low *versus* high Goutallier grades. BSC also showed a strong negative correlation with the supraspinatus occupation ratio.

## Supplementary information

**Additional file 1: Supplemental Fig. S1.** Diagram illustrating the processing steps required to compute the backscatter coefficient (BSC). Using an ultrasound probe (**a**), images of the rotator cuff muscle (**b**) and reference phantom with a known BSC (**c**) are captured. The raw radiofrequency (RF) data is collected and used to compute the power spectra (**d**-**e**) for the region of interest (ROI) that is manually outlined on the muscle and automatically propagated to the reference phantom image (outlined in white in **b** and **c**). Calibration of the data is achieved by computing a ratio from these spectra, and depth-dependent attenuation is compensated using the spectral log difference method on the ROI placed on the overlying tissues (outlined in orange in (**b**). BSC as a function of frequency is plotted (**f**), which is system-independent. **Supplemental Fig. S2.** Histogram distributions of backscatter coefficient values for Goutallier 0-2 and 3-4 grades for both supraspinatus (**a**) and infraspinatus (**b**) muscles. **Supplemental Fig. S3.** Receiver operating characteristic curves for the supraspinatus (**a**) and infraspinatus (**b**) muscles with 1,000-fold bootstrapping. Area under the curves (AUCs) with 95% confidence intervals (95% CIs, outlined in shaded areas in **a** and **b**) were 0.98 (0.93–1.0) for the supraspinatus and 0.98 (0.94–1.0) for the infraspinatus muscles.

## Data Availability

The datasets used and/or analyzed during the current study are available from the corresponding author upon reasonable request.

## Abbreviations

AUROC: Area under the receiver operating characteristic curve
BSC: Backscatter coefficient

## References

[1] Jancuska J, Matthews J, Miller T, Kluczynski MA, Bisson LJ (2018) A systematic summary of systematic reviews on the topic of the rotator cuff. Orthop J Sports Med 6:2325967118797891. 10.1177/2325967118797891 30320144 10.1177/2325967118797891 PMC6154263

[2] Brindisino F, Salomon M, Giagio S, Pastore C, Innocenti T (2021) Rotator cuff repair vs. nonoperative treatment: a systematic review with meta-analysis. J Shoulder Elbow Surg 30:2648–2659. 10.1016/j.jse.2021.04.040 34020002 10.1016/j.jse.2021.04.040

[3] Kuzel BR, Grindel S, Papandrea R, Ziegler D (2013) Fatty infiltration and rotator cuff atrophy. J Am Acad Orthop Surg 21:613–623. 10.5435/JAAOS-21-10-613 24084435 10.5435/JAAOS-21-10-613

[4] Tsuchiya S, Bois AJ, Matthewson G, Oiwa S, More KD, Lo IKY (2023) The relationship between preoperative Goutallier stage and retear rates following posterosuperior rotator cuff repair: a systematic review. J Shoulder Elbow Surg 32:435–443. 10.1016/j.jse.2022.09.011 36252788 10.1016/j.jse.2022.09.011

[5] Zoga AC, Kamel SI, Hynes JP, Kavanagh EC, O’Connor PJ, Forster BB (2021) The evolving roles of MRI and ultrasound in first-line imaging of rotator cuff injuries. AJR Am J Roentgenol 217:1390–1400. 10.2214/AJR.21.25606 34161130 10.2214/AJR.21.25606

[6] Middleton WD, Payne WT, Teefey SA, Hildebolt CF, Rubin DA, Yamaguchi K (2004) Sonography and MRI of the shoulder: comparison of patient satisfaction. AJR Am J Roentgenol 183:1449–1452. 10.2214/ajr.183.5.1831449 15505319 10.2214/ajr.183.5.1831449

[7] Sconfienza LM, Albano D, Allen G et al (2018) Clinical indications for musculoskeletal ultrasound updated in 2017 by European Society of Musculoskeletal Radiology (ESSR) consensus. Eur Radiol 28:5338–5351. 10.1007/s00330-018-5474-3 29876703 10.1007/s00330-018-5474-3

[8] Khoury V, Cardinal E, Brassard P (2008) Atrophy and fatty infiltration of the supraspinatus muscle: sonography versus MRI. AJR Am J Roentgenol 190:1105–1111. 10.2214/AJR.07.2835 18356462 10.2214/AJR.07.2835

[9] Wall LB, Teefey SA, Middleton WD et al (2012) Diagnostic performance and reliability of ultrasonography for fatty degeneration of the rotator cuff muscles. J Bone Joint Surg Am 94:e83. 10.2106/JBJS.J.01899 22717835 10.2106/JBJS.J.01899 PMC3368496

[10] Tenbrunsel TN, Whaley JD, Golchian D, Malone DL, Lima DJL, Sabesan VJ (2019) Efficacy of imaging modalities assessing fatty infiltration in rotator cuff tears. JBJS Rev 7:e3. 10.2106/JBJS.RVW.18.00042 30969180 10.2106/JBJS.RVW.18.00042

[11] Strobel K, Hodler J, Meyer DC, Pfirrmann CW, Pirkl C, Zanetti M (2005) Fatty atrophy of supraspinatus and infraspinatus muscles: accuracy of US. Radiology 237:584–589. 10.1148/radiol.2372041612 16192321 10.1148/radiol.2372041612

[12] Ashir A, Jerban S, Barrere V et al (2023) Skeletal muscle assessment using quantitative ultrasound: a narrative review. Sensors (Basel) 23:4763. 10.3390/s23104763 37430678 10.3390/s23104763 PMC10222479

[13] Cloutier G, Destrempes F, Yu F, Tang A (2021) Quantitative ultrasound imaging of soft biological tissues: a primer for radiologists and medical physicists. Insights Imaging 12:127. 10.1186/s13244-021-01071-w 34499249 10.1186/s13244-021-01071-w PMC8429541

[14] Wear KA, Han A, Rubin JM et al (2022) US backscatter for liver fat quantification: an AIUM-RSNA QIBA pulse-echo quantitative ultrasound initiative. Radiology 305:526–537. 10.1148/radiol.220606 36255312 10.1148/radiol.220606

[15] Wu Y, Barrere V, Ashir A et al (2024) High-frequency quantitative ultrasound imaging of human rotator cuff muscles: assessment of repeatability and reproducibility. Ultrason Imaging 46:56–70. 10.1177/01617346231207404 37981826 10.1177/01617346231207404 PMC11170563

[16] Fuchs B, Weishaupt D, Zanetti M, Hodler J, Gerber C (1999) Fatty degeneration of the muscles of the rotator cuff: assessment by computed tomography versus magnetic resonance imaging. J Shoulder Elbow Surg 8:599–605. 10.1016/s1058-2746(99)90097-6 10633896 10.1016/s1058-2746(99)90097-6

[17] Somerson JS, Hsu JE, Gorbaty JD, Gee AO (2016) Classifications in brief: Goutallier classification of fatty infiltration of the rotator cuff musculature. Clin Orthop Relat Res 474:1328–1332. 10.1007/s11999-015-4630-1 26584800 10.1007/s11999-015-4630-1 PMC4814439

[18] Thomazeau H, Rolland Y, Lucas C, Duval JM, Langlais F (1996) Atrophy of the supraspinatus belly. assessment by MRI in 55 patients with rotator cuff pathology. Acta Orthop Scand 67:264–268. 10.3109/17453679608994685 8686465 10.3109/17453679608994685

[19] Han A, Andre MP, Erdman JW, Loomba R, Sirlin CB, O’Brien WD (2017) Repeatability and reproducibility of a clinically based QUS phantom study and methodologies. IEEE Trans Ultrason Ferroelectr Freq Control 64:218–231. 10.1109/TUFFC.2016.2588979 27411218 10.1109/TUFFC.2016.2588979 PMC5283517

[20] Yao LX, Zagzebski JA, Madsen EL (1990) Backscatter coefficient measurements using a reference phantom to extract depth-dependent instrumentation factors. Ultrason Imaging 12:58–70. 10.1177/016173469001200105 2184569 10.1177/016173469001200105

[21] Labyed Y, Bigelow TA (2010) Estimating the total ultrasound attenuation along the propagation path by using a reference phantom. J Acoust Soc Am 128:3232–3238. 10.1121/1.3483739 21110618 10.1121/1.3483739 PMC3003735

[22] Labyed Y, Bigelow TA (2011) A theoretical comparison of attenuation measurement techniques from backscattered ultrasound echoes. J Acoust Soc Am 129:2316–2324. 10.1121/1.3559677 21476687 10.1121/1.3559677 PMC3087399

[23] Landis JR, Koch GG (1977) The measurement of observer agreement for categorical data. Biometrics 33:159–174 843571

[24] Schober P, Boer C, Schwarte LA (2018) Correlation coefficients: appropriate use and interpretation. Anesth Analg 126:1763–1768. 10.1213/ANE.0000000000002864 29481436 10.1213/ANE.0000000000002864

[25] Nahm FS (2022) Receiver operating characteristic curve: overview and practical use for clinicians. Korean J Anesthesiol 75:25–36. 10.4097/kja.21209 35124947 10.4097/kja.21209 PMC8831439

[26] Habibzadeh F, Habibzadeh P, Yadollahie M (2016) On determining the most appropriate test cut-off value: the case of tests with continuous results. Biochem Med (Zagreb) 26:297–307. 10.11613/BM.2016.034 27812299 10.11613/BM.2016.034 PMC5082211

[27] Gyftopoulos S, Guja KE, Subhas N, Virk MS, Gold HT (2017) Cost-effectiveness of magnetic resonance imaging versus ultrasound for the detection of symptomatic full-thickness supraspinatus tendon tears. J Shoulder Elbow Surg 26:2067–2077. 10.1016/j.jse.2017.07.012 28893546 10.1016/j.jse.2017.07.012

[28] Park BK, Hong SH, Jeong WK (2020) Effectiveness of ultrasound in evaluation of fatty infiltration in rotator cuff muscles. Clin Orthop Surg 12:76–85. 10.4055/cios.2020.12.1.76 32117542 10.4055/cios.2020.12.1.76 PMC7031432

[29] Wu Y, Barrere V, Han A, Chang EY, Andre MP, Shah SB (2023) Repeatability, reproducibility and sources of variability in the assessment of backscatter coefficient and texture parameters from high-frequency ultrasound acquisitions in human median nerve. Ultrasound Med Biol 49:122–135. 10.1016/j.ultrasmedbio.2022.08.007 36283940 10.1016/j.ultrasmedbio.2022.08.007

[30] Lippe J, Spang JT, Leger RR, Arciero RA, Mazzocca AD, Shea KP (2012) Inter-rater agreement of the Goutallier, Patte, and Warner classification scores using preoperative magnetic resonance imaging in patients with rotator cuff tears. Arthroscopy 28:154–159. 10.1016/j.arthro.2011.07.016 22019235 10.1016/j.arthro.2011.07.016

[31] Davis DL, Gilotra MN, Calderon R, Roberts A, Hasan SA (2021) Reliability of supraspinatus intramuscular fatty infiltration estimates on T1-weighted MRI in potential candidates for rotator cuff repair surgery: full-thickness tear versus high-grade partial-thickness tear. Skeletal Radiol 50:2233–2243. 10.1007/s00256-021-03805-9 33959799 10.1007/s00256-021-03805-9 PMC8565455

[32] Schiefer M, Mendonca R, Magnanini MM et al (2015) Intraobserver and interobserver agreement of Goutallier classification applied to magnetic resonance images. J Shoulder Elbow Surg 24:1314–1321. 10.1016/j.jse.2015.02.011 25940380 10.1016/j.jse.2015.02.011

[33] Ro K, Kim JY, Park H et al (2021) Deep-learning framework and computer-assisted fatty infiltration analysis for the supraspinatus muscle in MRI. Sci Rep 11:15065. 10.1038/s41598-021-93026-w 34301978 10.1038/s41598-021-93026-w PMC8302634

