# Supplementary material for: Quantitative ultrasound assessment of fatty infiltration of the rotator cuff muscles using backscatter coefficient
Source: Eur Radiol Exp. 2024 Oct 22;8:119. doi: 10.1186/s41747-024-00522-5 (PMC11496476; doi:10.1186/s41747-024-00522-5)
Supplement: Supplementary file 1 — Additional file 1: Supplemental Fig. S1. Diagram illustrating the processing steps required to compute the backscatter coefficient (BSC). Using an ultrasound probe (a), images of the rotator cuff muscle (b) and reference phantom with a known BSC (c) are captured. The raw radiofrequency (RF) data is collected and used to compute the power spectra (d-e) for the region of interest (ROI) that is manually outlined on the muscle and automatically propagated to the reference phantom image (outlined in white in b and c). Calibration of the data is achieved by computing a ratio from these spectra, and depth-dependent attenuation is compensated using the spectral log difference method on the ROI placed on the overlying tissues (outlined in orange in (b). BSC as a function of frequency is plotted (f), which is system-independent. Supplemental Fig. S2. Histogram distributions of backscatter coefficient values for Goutallier 0-2 and 3-4 grades for both supraspinatus (a) and infraspinatus (b) muscles. Supplemental Fig. S3. Receiver operating characteristic curves for the supraspinatus (a) and infraspinatus (b) muscles with 1,000-fold bootstrapping. Area under the curves (AUCs) with 95% confidence intervals (95% CIs, outlined in shaded areas in a and b) were 0.98 (0.93–1.0) for the supraspinatus and 0.98 (0.94–1.0) for the infraspinatus muscles. [file 41747_2024_522_MOESM1_ESM.pdf]

Quantitative ultrasound assessment of fatty infiltration of the rotator cuff muscles using backscatter coefficient

ELECTRONIC SUPPLEMENTARY MATERIAL

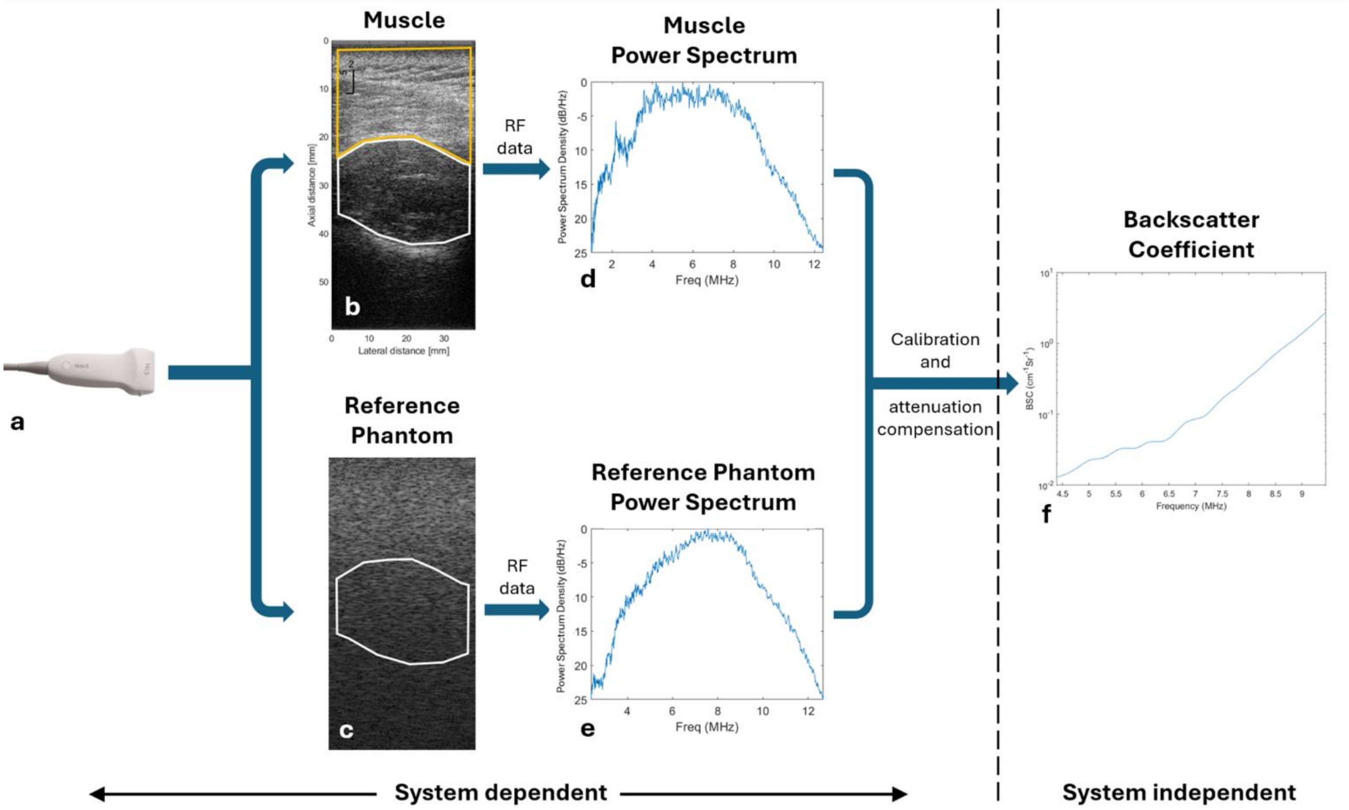

Supplemental Fig. S1. Diagram illustrating the processing steps required to compute the backscatter coefficient (BSC). Using an ultrasound probe (a), images of the rotator cuff muscle (b) and reference phantom with a known BSC (c) are captured. The raw radiofrequency (RF) data is collected and used to compute the power spectra (d-e) for the region of interest (ROI) that is manually outlined on the muscle and automatically propagated to the reference phantom image (outlined in white in b and c). Calibration of the data is achieved by computing a ratio from these spectra, and depth-dependent attenuation is compensated using the spectral log difference method on the ROI placed on the overlying tissues (outlined in orange in b). BSC as a function of frequency is plotted (f), which is system independent.

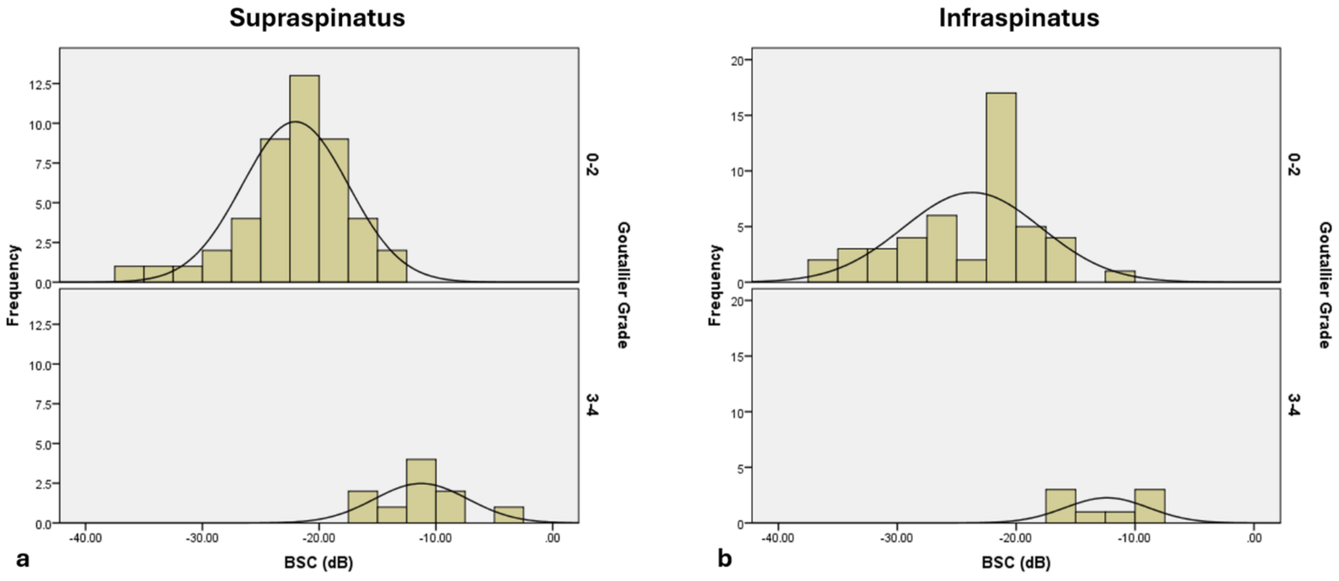

Supplemental Fig. S2. Histogram distributions of backscatter coefficient values for Goutallier 0-2 and 3-4 grades for both supraspinatus (a) and infraspinatus (b) muscles. *BSC* backscatter coefficient.

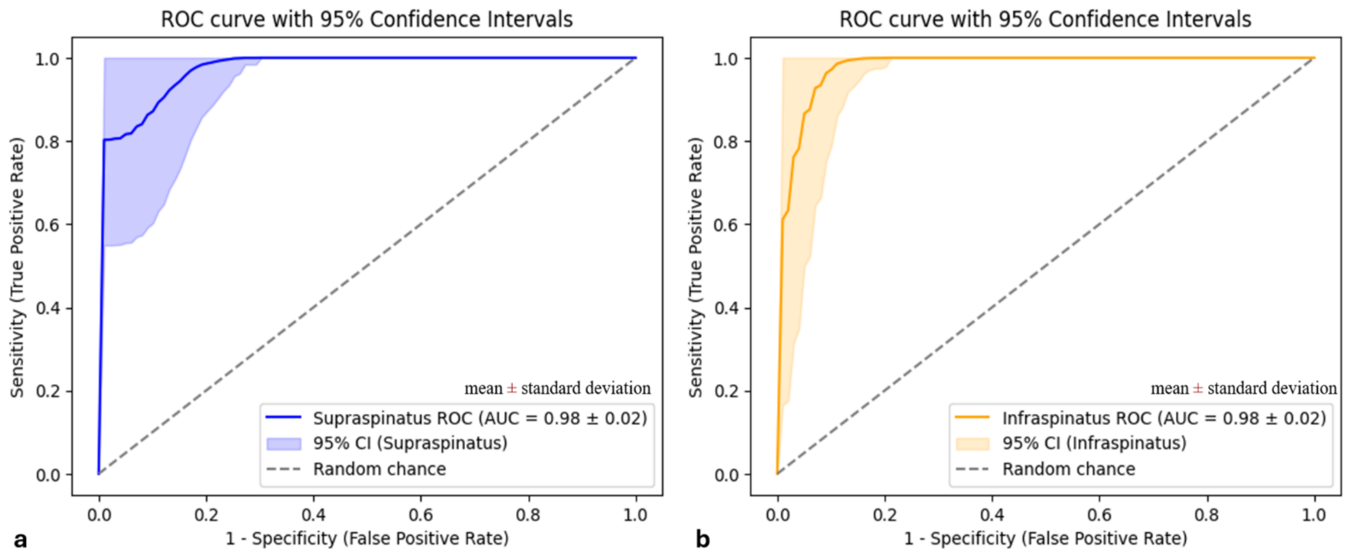

Supplemental Fig. S3. Receiver operating characteristic curves for the supraspinatus (a) and infraspinatus (b) muscles with 1,000-fold bootstrapping. Area under the curves (AUCs) with 95% confidence intervals [95% CIs, outlined in shaded areas in a and b] were 0.98 [0.93 - 1.0] for the supraspinatus and 0.98 [0.94 - 1.0] for the infraspinatus muscles.
